# Why Cognitive Behavioral Therapy Is the Current Gold Standard of Psychotherapy

**DOI:** 10.3389/fpsyt.2018.00004

**Published:** 2018-01-29

**Authors:** Daniel David, Ioana Cristea, Stefan G. Hofmann

**Affiliations:** ^1^Department of Clinical Psychology and Psychotherapy, International Institute for Advanced Study in Psychotherapy and Applied Mental Health at Babes-Bolyai University, Cluj-Napoca, Romania; ^2^Department of Population Health Sciences and Policy at Icahn School of Medicine at Mount Sinai, New York, NY, United States; ^3^Department of Psychological and Brain Sciences, Boston University, Boston, MA, United States

**Keywords:** cognitive behavioral therapy, gold standard, evidence-based practices, informed decision-making, guidelines

Taking into account the number of publications/studies, academic programs, and/or practicing professionals, cognitive behavioral therapy (CBT) is arguably the *gold standard* of the psychotherapy field. However, recently, some colleagues have argued for plurality in psychotherapy, questioning the status of CBT as the *gold standard* in psychotherapy (1), because many studies are of low quality and/or the comparator conditions are weak (i.e., wait list rather than active comparators), thus challenging CBT’s prominent status among academic programs and practitioners.

We think that many issues factor into the *gold-standard* designation. If *gold standard* is defined as best standard we can have in the field, then, indeed, CBT is not the *gold standard*, and CBT, as a progressive research program, would not even argue for such a status at this moment. However, if *gold standard* is defined as best standard we have in the field at the moment, then we argue that CBT is, indeed, the *gold standard*.

In this paper, we argue that CBT is the *gold-standard* psychological treatment—as the best standard we have in the field currently available—for the following reasons [see also Hofmann et al. (2)]: (1) CBT is the most researched form of psychotherapy. (2) No other form of psychotherapy has been shown to be systematically superior to CBT; if there are systematic differences between psychotherapies, they typically favor CBT. (3) Moreover, the CBT theoretical models/mechanisms of change have been the most researched and are in line with the current mainstream paradigms of human mind and behavior (e.g., information processing). At the same time, there is clearly room for further improvement, both in terms of CBT’s efficacy/effectiveness and its underlying theories/mechanisms of change. We further argue for an integrated scientific psychotherapy, with CBT serving as the foundational platform for integration.

Modern CBT is an umbrella term of empirically supported treatments for clearly defined psychopathologies that are targeted with specific treatment strategies (3). More recently, CBT has included a more trans-diagnostic/process-based and personalized approach, with the ultimate goal of linking the therapeutic technique to the process and the individual client (4). Traditionally, clinical trials examining the efficacy of CBT include waitlist control, placebo conditions, treatment as usual/TAU, and other alternative treatments (including psychodynamic therapies and pharmacotherapies).

Although a number of CBT trials have included weak comparisons (e.g., wait list control conditions), there are also many studies that compared CBT to strong comparison conditions (e.g., pill or psychological placebo, TAU, other psychotherapies, pharmacotherapy), meeting the stringent criteria of an empirically supported treatment (5). Indeed, Cuijpers et al. (6) found that about 54% of total trials for depression (about 34 trials) and about 20% of total trials for anxiety (about 25) met the criteria for a strong comparison (i.e., pill placebo or TAU). Cuijpers et al. (6) further reported that 17% of the total trials for depression and anxiety were of high quality and that the relationship between the quality of CBT studies and the effect sizes was not strong. Most psychotherapies [e.g., except only interpersonal therapy for depression (7), which has similar numbers] do not even come close to these numbers in terms of the active status of the comparator and the study quality [see the case of psychodynamic therapies for depression (8) and anxiety (9)]. When compared to TAU or various active conditions CBT often has a small/moderate (for TAU) or small/no effect (for active conditions). However, in these conditions, even a small effect size might be very important clinically (10), depending on the cost and benefit analyses as well as if it is cumulative or not (e.g., in time and/or population).

Cognitive behavioral therapy was the first form of psychotherapy tested with the most stringent criteria (e.g., randomized trials and active comparator) of evidence-based framework used in the health field (e.g., similar for those used in case of pharmacotherapy). Therefore, it was the first psychotherapy largely identified as evidence-based in most clinical guidelines (along with interpersonal psychotherapy for depression). Consequently, many newer, less thoroughly and/or later tested psychotherapies started to use CBT as the reference treatment, often arguing for their efficacy/effectiveness when finding no difference from CBT. However, no difference to CBT can be invoked as support for a kind of clinical similarity only in equivalence or non-inferiority designs, not in superiority designs (and many of such comparisons were not framed as equivalence/non-inferiority designs). Moreover, statistically speaking, if B is equivalent to A and C is equivalent to B, it is not guaranteed that C will be also equivalent to A. Thus, if therapy A is the reference treatment and one proves that psychotherapy B is equivalent to A, it does allow psychotherapy B to become a reference treatment for the test of a new psychotherapy C. For example, Steinert et al. (11) conducted an equivalence meta-analysis for psychodynamic psychotherapies (PP) with the existing *gold standard* (most of the time CBT) and found the equivalence to be supported for the interval −0.25 to +0.25. However, equivalence is not transitive. If B (PP) is equivalent to the *gold-standard* A (i.e., CBT), it does not mean that B could be used as a *gold standard* for a new treatment C, as the equivalence between B and C does not imply the equivalence between A and C. This transitivity is even problematic in this case because, in the equivalence limit, significant differences (for 90% Equivalence CI) favoring *gold standard* over PP were found for (1) target symptoms (posttreatment: *g* = −0.158; *k* = 21) and (2) general psychiatric symptoms (*g* = −0.116; *k* = 15). Thus, even if the equivalence of PP to CBT was supported, it does not mean that PP gains the same reference status as CBT. Instead, PP should independently pass the same tests as the *gold standard* to obtain the same status (e.g., several high quality independent clinical trials using placebo or other active comparators).

Concerning theory/mechanisms of change, CBT is (1) integrated in the larger mainstream information processing paradigm, where the causal role of explicit or implicit cognitions in generating emotions and behaviors is already well-established [although various cognitions targeted by CBT have different research-based support (3)], (2) continuously evolving based on both cumulative and critical research (12), and (3) integrated into a larger picture of science (e.g., cognitive neurogenetics). At this moment, there are no other psychological treatments with more research support to validate their underlying constructs. In contrast, some psychological treatments—especially those derived from classical psychoanalysis—are unsupported or controversial with regards to the underlying constructs,^1^ while others (e.g., interpersonal psychotherapy) are in an incipient phase (13).

In summary, because of its clear research support, CBT dominates the international guidelines for psychosocial treatments, making it a first-line treatment for many disorders, as noted by the National Institute for Health and Care Excellence’s guidelines^2^ and American Psychological Association.^3^ Therefore, CBT is, indeed, the *gold standard* in the psychotherapy field, being included in the major clinical guidelines based on its rigorous empirical basis, not for various political reasons, as some colleagues (1) seem to suggest. Having said that, we must add that, although CBT is efficacious/effective, there is still room for improvement, as in many situations there are patients who do not respond to CBT and/or relapse. While many non-CBT psychotherapies have changed little in practice since their creation, CBT is an evolving psychotherapy based on research (i.e., a progressive research program). Therefore, we predict that continuous improvements in psychotherapy will derive from CBT, gradually moving the field toward an integrative scientific psychotherapy.

## Author Note

A longer quantitative form of the present viewpoint is under preparation.

## Author Contributions

DD, IC, and SH substantially contributed to the conception of the work, drafting different components of the manuscript and revising other components. All authors approved the submitted version of e manuscript and agreed to be accountable for all aspects of the work.

## Conflict of Interest Statement

SH receives compensation for his work as an advisor from the Palo Alto Health Sciences and for his work as a Subject Matter Expert from John Wiley & Sons, Inc. and SilverCloud Health, Inc. He also receives royalties and payments for his editorial work from various publishers. DD receives consultation fee from the Albert Ellis Institute and editorial fee from the Springer. All three authors are CBT trained scientists, active promoters, and contributors to evidence-based psychotherapy.

## Footnotes

^1^https://www.div12.org/psychological-treatments/treatments/psychoanalytic-treatment-for-panic-disorder/.

^2^http://www.nice.org.uk.

^3^https://www.div12.org/psychological-treatments/.
